# Evolving therapeutic strategies in mantle cell lymphoma: advancements and future directions

**DOI:** 10.1038/s41375-026-02942-1

**Published:** 2026-04-15

**Authors:** Rita Tavarozzi, Nawar Maher, Gioacchino Catania, Giulia Zacchi, Francesca D’Andrea, Antonella Sofia, Manuela Zanni, Marco Ladetto

**Affiliations:** 1 Department of Translational Medicine, University of Eastern Piedmont, Novara, Italy; 2 SCDU of Hematology, Azienda Ospedaliera Universitaria SS Antonio e Biagio e Cesare Arrigo, Alessandria, Italy

**Keywords:** B-cell lymphoma, Clinical trials, Drug development, Cancer therapy

## Abstract

Mantle cell lymphoma (MCL) is a biologically and clinically heterogeneous B-cell malignancy with a historically poor prognosis. Recent advances have substantially expanded treatment options, particularly through the integration of targeted therapies, chemotherapy-free regimens, and cellular approaches. Frontline treatment now incorporates Bruton’s tyrosine kinase inhibitors (BTKi) in combination with chemotherapy and in chemotherapy-free regimens. For patients with relapsed or refractory disease, particularly those previously exposed to BTKi, chimeric antigen receptor T-cell (CAR T) therapies and third-generation BTKi like pirtobrutinib provide highly effective options while several novel agents, including bispecific antibodies (bsAbs), are under investigation. Nevertheless, achieving long-lasting remissions is still a major challenge, especially among high-risk patients. Future directions include optimizing sequencing, refining rational combination therapies, expanding the application of bsAbs, and integrating small molecules and novel immunoconjugates to enhance therapeutic efficacy and long-term outcomes. This review provides an overview of the current and emerging therapies for MCL, highlighting how the integration of biological agents, strategic combinations, and patient-centered approaches are driving the next phase of MCL treatment.

## Introduction

Mantle cell lymphoma (MCL) is an uncommon and aggressive subtype of B-cell non-Hodgkin lymphoma (B-NHL), first recognized as a distinct clinicopathological entity in the 1980s and formally classified by the World Health Organization (WHO) in 1994 [1, 2]. It is mainly characterized by the t(11;14) translocation, which leads to cyclin D1 overexpression and uncontrolled cell proliferation [3, 4].

MCL presents with a broad clinical spectrum, ranging from indolent, slow-progressing to highly aggressive forms [3, 4]. While in the past MCL was associated with poor outcomes, with a median survival of just 3–5 years [2], advancements in risk stratification, and treatment have significantly improved the outcomes of MCL patients [5].

This review examines the recent therapeutic progress in MCL, with a particular focus on targeted therapies and emerging immunotherapeutic approaches. Ultimately, it aims to provide clinicians with an updated framework to understand the ongoing therapeutic advances and guide informed, patient-centered decision-making.

## Prognostic features In MCL

As the therapeutic landscape continues to evolve, a deeper understanding of the prognostic factors influencing MCL outcome has become increasingly important. Prognostic models now integrate histopathologic characteristics [3, 4, 6], clinical indices [7–10], genomic alterations [11–13], relapse timing [14], and measurable residual disease (MRD) [15–19] status. While a comprehensive description of these factors goes beyond the scope of this review, we herein summarize the key clinical predictors currently identified (Table 1), along with a critical evaluation of their limitations (Table 2).

**Table 1:** Selected clinical prognostic scores in MCL.

Scoring system	Components	Calculation	Risk group	PFS	OS
MIPI [7]	Age, ECOG PS, LDH, WCC	0.03535 × age (years) + 0.6978 (if ECOG PS > 1) + 1.367 × log_10_ (LDH/ULN) + 0.9393 × log_10_ (WCC per 10^-6^)	LR < 5.70 IR ≥ 5.70 HR ≥ 6.20		5-year OS LR 83% IR 63% HR 34%
s-MIPI [8]	Age, ECOG PS, LDH, WCC	- Age: 1 point for 50–59, 2 for 60–69, 3 for ≥ 70 years - ECOG: 2 points for ECOG 2-4 - LDH/ULN ratio: 1 point for 0.67–0.99, 2 points for 1.0–1.49, 3 points for ≥ 1.50 - WCC 10^9^/L: 1 point 6.7–9.99, 2 points 1.00–14.99, 3 points ≥ 15.0	LR: 0–3 IR: 4–5 HR: 6–11	LR: not reached IR: 58 months HR: 37 months	LR 60% IR 35% HR 20%
MIPI-b [9]	Age, ECOG PS, LDH, WCC, Ki67%	CBS: 0.03535 × age (years) 1 0.6978 (if ECOG > 1) + 1.367 × log10(LDH/ULN) + 0.9393 x log10(WCC) + 0.02142 × Ki67%.	LR: CBS < 5.7 IR: CBS 5.7–6.5 HR: CBS ≥ 6.5	3-year PFS LR 74.9% IR 43.4% HR 16.1%	3-year OS LR84.4%, IR62.2%, HR 27.6%
MIPI-c [10]	Age, ECOG PS, LDH, WCC, Ki67%	- MIPI calculation - Ki67% ( < 30% or ≥ 30%)	LR: LR MIPI + Ki67 < 30% LI: either LR MIPI + Ki67 ≥ 30% or IR MIPI + Ki67 < 30% HI: either IR MIPI + Ki67 ≥ 30% or HR MIPI + Ki67 < 30% HR: HR MIPI and Ki67 ≥ 30%		5-year OS LR 85% LI 72% HI 43% HR 17%

*Age* Patient age (years), *CBS* Continuous Biological Score, *ECOG PS* Eastern Cooperative Oncology Group Performance Status,

*HI* High-Intermediate Risk, *HR* High Risk, *IR* Intermediate Risk, *Ki67%* Ki-67 proliferation index (percentage of tumor cells staining positive), *LDH* Lactate Dehydrogenase, *LI* Low-Intermediate Risk, *LR* Low Risk, *MIPI* Mantle Cell Lymphoma International Prognostic Index, *MIPI-b* Biologic Mantle Cell Lymphoma International Prognostic Index, *MIPI-c* Combined Mantle Cell Lymphoma International Prognostic Index, *OS* Overall Survival, *PFS* Progression-Free Survival, *s-MIPI* Simplified Mantle Cell Lymphoma International Prognostic Index, *ULN* Upper Limit of Normal, *WCC* White Cell Count (white blood cell count).

**Table 2:** Strengths/utility and limitations / pitfalls of the most useful prognosticators in MCL.

Prognostic factor (s)	Strengths / utility	Limitations / pitfalls
**MIPI / MIPIs/ MIPI-b / MIPI-c**	Widely validated; simple clinical variables; refined by Ki-67 to improve discrimination.	Does not capture genomic complexity; risk groups still heterogeneous; limited predictive value for novel therapies.
**Timing of relapse (POD24)**	Robust predictor of poor outcome; independent of other indices; easy to assess.	Post-hoc metric (only assessable after treatment); does not inform baseline therapy choice; few effective interventions for early POD24.
**Immunohistochemistry (Ki-67, TP53 expression)**	Readily available in routine pathology correlates with survival.	Ki-67 interpretation variable; TP53 expression not a reliable surrogate for TP53 mutation; semi-quantitative.
**Genomics (TP53, complex karyotype)**	TP53 mutation is one of the strongest adverse predictors; genomic profiling refines risk beyond clinical scores.	Requires access to NGS; cost and availability vary; 17p deletion alone insufficient; complexity of interpretation.
**Measurable residual disease (MRD)**	Strong predictor of relapses and survival; applicable across therapies (chemo, BTKi, CAR-T); dynamic monitoring possible.	Standardization lacking; assays vary (ASO-PCR, qPCR, NGS); limited routine uses outside clinical trials; uncertain how best to guide therapy in practice.

*17p* Short arm of chromosome, 17, *ASO-PCR* Allele-Specific Oligonucleotide Polymerase Chain Reaction, *BTKi* Bruton Tyrosine Kinase inhibitor, *CAR-T* Chimeric Antigen Receptor T-cell therapy, *Ki-67* Ki-67 proliferation index, *MIPI* Mantle Cell Lymphoma International Prognostic Index, *MIPI-b* Biologic Mantle Cell Lymphoma International Prognostic Index, *MIPI-c* Combined Mantle Cell Lymphoma International Prognostic Index, *MIPIs* Mantle Cell Lymphoma International Prognostic Indexes (plural), *MRD* Minimal Residual Disease, *NGS* Next-Generation Sequencing, *POD24* Progression of Disease within 24 months, *qPCR* Quantitative Polymerase Chain Reaction, *TP53* Tumor Protein 53 gene.

## First-Line Treatment Of Mcl

### The historical backbone in MCL

For many years, chemoimmunotherapy (CI) represented the standard first-line approach for patients with previously untreated MCL [20–25], although its role was increasingly being challenged by emerging biologic and targeted strategies [26, 27]. In younger, fit patients, Ara-C-containing induction followed by autologous stem-cell transplantation (ASCT) was historically considered the conventional standard [20–22]; however, accumulating evidence showed that the clinical benefit of ASCT was substantially diminished [28, 29].

For patients who are ineligible for intensive therapy, there was no universally accepted front-line treatment [24, 25, 30]. Bendamustine-based regimens were widely used due to their favorable balance of efficacy and tolerability. R-bendamustine (R-B) achieved higher response rates and longer PFS compared with R-CHOP, and in day-to-day practice many clinicians preferred R-B because of its superior tolerability profile, especially regarding neuropathy and alopecia [23, 24]. The low-dose cytarabine–containing R-BAC regimen produced deep and durable responses in older patients, and in experienced centers it was often considered for fit elderly individuals able to tolerate the added myelosuppression [25]. VR-CAP provided modest PFS improvement over R-CHOP, but its increased hematologic toxicity and neuropathy limited its uptake [30]; clinically, it was typically reserved for patients in whom maximizing cytotoxic intensity was prioritized and where close toxicity monitoring was feasible.

Rituximab maintenance (RM) after ASCT prolonged PFS and remained standard for younger patients [22]. In the non-transplant setting, several studies supported RM following conventional CI, and in practice many clinicians considered RM as a low-burden intervention capable of meaningfully extending remission in older or less-fit individuals [31]. Lenalidomide alone did not show comparable benefit and was rarely used for maintenance outside clinical trials [32]. The MCL-R2 Elderly trial demonstrated that rituximab-lenalidomide (R2) maintenance after cytarabine-based induction improved PFS in patients over 60, but the additional toxicity, particularly cytopenias and infections [33], has made clinicians cautious, and its application is generally limited to carefully selected patients.

Overall, these developments underscored the practical limitations of conventional CI where toxicity, durability of response, and patient preferences strongly influence treatment selection. They also highlighted the growing need for more personalized frontline strategies, particularly those integrating target agents, cellular therapies which are increasingly shaping contemporary clinical practice.

### Advancements in first-line treatments

#### Integration of BTKi in chemotherapy backbones

The TRIANGLE study has fundamentally reshaped frontline management for younger patients with MCL who were candidates for HDT [26]. The three-arm design directly compared conventional ASCT-based therapy with two fixed-duration, ibrutinib-containing strategies, including one that entirely omitted ASCT. With more than four years of follow-up, adding ASCT to an ibrutinib-based regimen (A + I) did not improve failure-free survival (FFS). Nevertheless, both ibrutinib-containing arms clearly outperformed ASCT alone. The 3-year overall survival (OS) of 90–91% in the ibrutinib arms versus 85% with standard therapy represents a clinically meaningful benefit that is difficult to ignore [34]. From a practical standpoint, these data demonstrates that ibrutinib, not transplant, drives improved outcomes in this population.

Toxicity patterns also influence real-world decision-making. Although ibrutinib did not increase severe events during induction or ASCT, combining both modalities (A + I) predictably raised the risk of hematologic toxicity and infections during maintenance [35]. This reinforces the idea that the lack of value of HDT is limited once effective targeted therapy is incorporated.

TRIANGLE also provided a valuable retrospective look at RM [35]. Across all treatment groups, RM consistently improved PFS even in the BTKi era. Although higher rates of grade ≥3 infections were reported, infection-related mortality did not increase [35]. RM thus remains a low-burden, clinically meaningful post-induction strategy.

For older or transplant-ineligible patients, BTKi-based frontline therapy is similarly associated with meaningful benefit, although the evidence has matured differently, with several limitations particularly due to indefinite BTKi-maintenance strategy [27, 36].

The SHINE trial, which added continuous ibrutinib to a BR induction backbone, demonstrated a substantial prolongation of PFS but the lack of an OS advantage [37], along with higher rates of atrial fibrillation, infections, and cytopenias, has led many clinicians to adopt a cautious, selective approach to BR + I. In real-world practice, cardiovascular toxicity remains a major barrier to the broad adoption of this regimen in elderly patients.

In contrast, the ECHO trial offers a more compelling, practice-changing BTKi–chemo frontline option for this patient population. Continuous acalabrutinib added to a BR-induction–based strategy significantly improved PFS, with a safety profile that was markedly more manageable than that observed in SHINE. A 50-month follow-up analysis further confirmed the durability of the ABR regimen, demonstrating a 24% reduction in the risk of initiating next-line therapy or death [36]. Acalabrutinib’s lower cardiac toxicity, reduced off-target effects, and overall better tolerability make it a far more practical option for elderly patients [27]. The updated analysis also reaffirmed the sustained PFS benefit and showed no emerging long-term safety concerns with acalabrutinib maintenance [36], supporting both the efficacy and long-term feasibility of ABR as a frontline option in treatment-naïve, non–HDT-eligible MCL patients, despite the potential limitations of indefinite maintenance therapy.

Taken together, TRIANGLE for young patients and ECHO for elderly patients have rewritten the frontline therapeutic landscape of MCL. They collectively mark the transition from a chemotherapy- and transplant-dominated paradigm to one in which BTK inhibitors are the cornerstone across age groups, with ASCT increasingly optional and chemo intensity adjusted to a patient’s biology and comorbidities [38].

The field is now clearly moving toward more targeted, better-tolerated, and more individualized approaches, bringing the promise of durable remissions closer to both young and elderly patients with MCL.

#### Integration of other biological agents in chemotherapy backbones

**Fig. 1:**
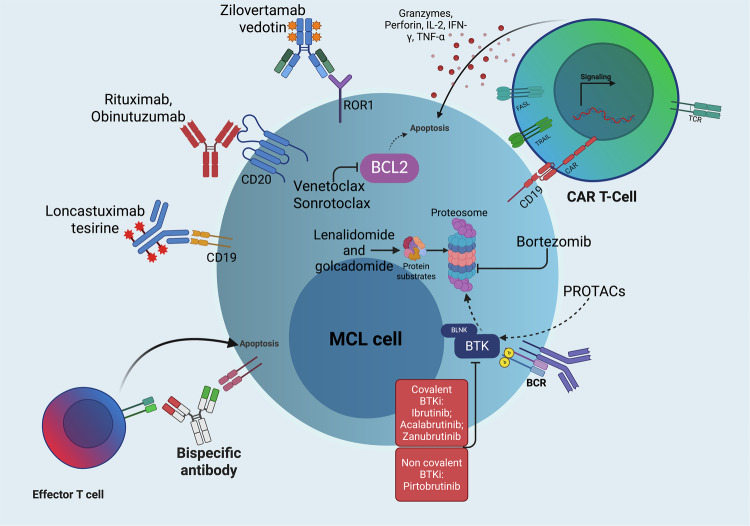
Novel agents in MCL. Created with Biorender.com.

Beyond BTK inhibitors, frontline exploration of BCL-2 inhibition has begun to take shape (Fig. 1) [39]. The FIL VR-BAC was the first study evaluating venetoclax added to bendamustine, rituximab, and cytarabine in older MCL patients, using a risk-adapted design that intentionally targeted those with adverse biology, precisely the group in whom conventional CI often falls short. The addition of venetoclax showed encouraging activity in high-risk subsets, with a toxicity profile that, while marked by expected cytopenias and infections, remained broadly manageable [39].

Although the trial does not yet position venetoclax as a frontline standard, it provides early proof that BCL-2 inhibition can be successfully integrated into intensive induction and may offer value for patients with biologically aggressive diseases.

#### Chemotherapy-free approaches

Frontline chemotherapy-free strategies are increasingly transforming the management of MCL, particularly in older patients or those unfit for intensive chemotherapy. Early proof-of-concept studies, such as the R2 phase II trial [40], demonstrated that rituximab-lenalidomide induction followed by maintenance achieved an overall response rate (ORR) of approximately 92% with a complete (CR) rate of about 64%, with durable responses and manageable safety profile [40]. Although this represents a limited experience, it provided a concept for chemotherapy-free approaches.

In the ENRICH trial, ibrutinib plus rituximab achieved high overall and complete response rates with superior PFS compared to R-CHOP, though not compared to BR. Cardiovascular toxicity, notably atrial fibrillation was reported in 7% of I-containing arm patients [41].

Notably, ENRICH represents one of the few randomized phase 3 trials evaluating a chemotherapy-free approaches in the frontline setting, underscoring the importance of randomized evidence in defining new standards of care.

Acalabrutinib and zanubrutinib, have emerged as more attractive partners for chemotherapy-free regimens due to its improved selectivity and safety. Preliminary clinical experience suggests that these agents were effective as first-generation ibrutinib while substantially reducing off-target toxicities, particularly cardiovascular events, which are a key concern in elderly patients. However, most data derive from early-phase or non-randomized studies, highlighting the need for confirmatory randomized trials.

The ongoing MANGROVE trial is evaluating until progression zanubrutinib plus rituximab schedule versus B-R in previously untreated patients, without RM, and its results are expected to clarify whether second-generation BTK inhibitors can safely replace standard CI in the frontline setting [42]. However, the study does not include RM, representing a key limitation in assessing the long-term durability of responses.

Chemotherapy-free triplet strategies are generating enthusiasm, especially for high-risk populations, such as *TP53*-mutated MCL. Early-phase studies, including OASIS (ibrutinib-obinutuzumab-venetoclax) [43], acalabrutinib-venetoclax-rituximab (AVR) [44], acalabrutinib-lenalidomide-rituximab (ALR) [45], and BOVEN (zanubrutinib-obinutuzumab-venetoclax) [46], have reported ORRs of 86–100%, high complete response rates, and 1-year PFS consistently above 85%. Toxicities were generally manageable, mainly neutropenia and mild gastrointestinal effects, with few treatment discontinuations [43–46]. These regimens may offer deep remissions even in biologically adverse disease and represent a promising chemotherapy-free option for fit elderly patients or those with high-risk molecular features [46]. However, larger studies and possible phase 3 confirmatory trials will be needed to reach definitive conclusions.

Recent data suggest that cellular therapies may also integrate into chemotherapy-free or low-intensity frontline approaches for very high-risk disease. In a single-center pilot study (WINDOW-3), brexucabtagene autoleucel (brexu-cel) used as consolidation after acalabrutinib–rituximab induction treatment achieved high rates of undetectable MRD in previously untreated high-risk MCL [47]. Although promising, the monocentric design, small sample size, and occurrence of high-grade CRS/ICANS, like relapsed/refractory settings, underscore the need for caution and longer follow-up.

Ongoing multicenter trials, such as CARMAN [48] will be essential to validate the feasibility and true impact of early CAR T-cell integration in frontline high-risk MCL and determine whether such strategies can meaningfully alter outcomes in this biologically adverse subgroup.

Taken together, these findings highlight a rapidly evolving paradigm in frontline MCL management. Yet most evidence remains early-phase, and longer follow-up is required to confirm durability, long-term safety, and optimal patient selection. Clinicians must weigh efficacy, tolerability, and biological risk while integrating emerging targeted therapies. With third-generation BTK inhibitors on the horizon, the frontline landscape is likely to continue evolving toward safer, highly effective, and chemotherapy-free strategies across age and risk groups (Table 3).

**Table 3:** Selected first-line clinical trials.

				Non-risk-adapted strategy								
				Targeted Combinations (BTKi + anti-CD20 ± CT)								
Study / NCT	BTKi	Anti-CD20	Third Agent	Phase / Design	n (pts)	Median Age (yrs)	Follow-up (mo)	PFS (mo)	OS	Inclusion Criteria	Risk profile (TP53, blastoid/pleomorphic, Ki-67 ≥ 30%)	Most relevant Grade ≥ 3 Toxicities (≥10%, per trial reports)
**SHINE** [37] **– NCT01776840**	Ibrutinib	Rituximab	CT	III, BR ± I → R-maintenance	523 (arm I vs arm placebo: 261 vs 262)	71	84.7	mPFS, arm I vs arm placebo: 80.6 vs 52.9 (p = 0.011)	7 yrs-OS, arm IBR vs arm placebo: 57% vs 55%	Elderly (65 yrs of age or older)	All-comers: HR allowed (arm I vs arm placebo: 10% vs 9%; 7% vs 10%; NA)	Neutropenia, thrombocytopenia, pneumonia, atrial fibrillation, hypertension, diarrhea (arm I vs arm placebo: 47% vs 48%; 12% vs 13%; 20% vs 14%; 13.9% vs 6.5%; 13.5% vs 11%; 46% vs 37%)
**ECHO** [27, 36] **– NCT02972840**	Acalabrutinib	Rituximab	CT	III, BR ± A → R-maintenance	598 (arm A vs arm placebo: 299 vs 299)	71	60.8	mPFS, arm A vs arm placebo: 72.5 vs 47.8 (p = 0.002)	NR	Elderly (65 yrs of age or older)	All-comers: HR allowed (arm A vs arm placebo: 7% vs 10%; 14% vs 13%; 47% vs 49%)	Neutropenia, anemia, infections, COVID-19 disease, atrial fibrillation, hypertension, diarrhea (arm A vs arm placebo: 35% vs 37%; 20% vs 10%; 78% vs 71%; 31% vs 21%; 6.7% vs 4.4%; 13.5% vs 11%; 37% vs 28%)
**ENRICH** [41] **– NCT11038174**	Ibrutinib	Rituximab	CT	II/III, R-CHOP/BR vs I-R	397 (arm IR vs arm CIT: 199 vs 198)	74	47.9	mPFS, arm IR vs arm CIT: 65.3 vs 42.4 (p = 0.003)	5 yrs OS, arm IR vs arm CIT: 58% vs 55%	Elderly (65 yrs of age or older)	All-comers: HR allowed (arm IR vs arm CIT: 28% vs 24%; 6% vs 8%; 39% vs 45%)	Neutropenia, infections, atrial fibrillation, hypertension, bleeding (arm IR vs arm CIT: 9% vs 19%; 12% vs 15%; 7% vs 1%; 11% vs 1–4%; 6% vs 3%)
**TRIANGLE** [21, 35] **– NCT02858258**	Ibrutinib	Rituximab	CT	III, R-CHOP/R-DHAP ± I ± ASCT	870 (arm A vs arm A + I vs arm I: 288 vs 292 vs 290)	57	53	4 y FFS: A + I 82% vs A 70% (p = 0.0026)	4 y OS 81–90%	ASCT-eligible, younger	All-comers: HR allowed (arm A vs arm A + I vs arm I: 11% vs 14% vs 16%; 11% vs 13% vs 12%; 33% vs 31% vs 32%)	Myelosuppression, infections, GI toxicity (arm A vs arm A + I vs arm I: 21% vs 50% vs 28%; 13% vs 25% vs 19%; 3% vs 6% vs 4%)
**ECOG-EA4181** [29] **NCT04115631**	Acalabrutinib	Rituximab	CT	III, BR/cytarabine-R ± A	359 (arm A vs B vs C vs D: 257, 259, 49, 85)	61	27.9	3 yrs PFS, arm vs arm B:77 vs 77%	3 yrs OS, arm A vs B: 82% vs 86%	All-comers (18-70 yrs)	All-comers: HR allowed - data not shown	-
		**Immunotherapy / Targeted combinations (BTKi + anti-CD20 + other agent)**										
**Study / NCT**	**BTKi**	**Anti-CD20**	**Third agent**	**Phase / design**	**n (pts)**	**Median Age (yrs)**	**Follow-up (mo)**	**PFS**	**OS**	**Inclusion Criteria**	**Risk profile (TP53, blastoid/pleomorphic, Ki-67** ≥ **30%)**	**Most relevant Grade** ≥ **3 Toxicities (≥10%)**
**OASIS II** [43] **– NCT04802590**	Ibrutinib	Obinutuzumab / Rituximab	Venetoclax	II, 42 cycles OR; I ± V for 24 cycles	51/arm	65	13.5	2-yrs PFS 88%	2-yrs OS 92%	All-comers (18-70 yrs)	All-comers: HR allowed (small numbers)	Neutropenia (arm A vs arm B: 11.8% vs 34%)
**AVR** [44]**– NCT05951959**	Acalabrutinib	Rituximab	Venetoclax	II, R until PD or ≤15 cycles; A until PD; V until C25	108	69	10.8	6-mo PFS 97%	6-mo OS 98.1%	All-comers ( ≥ 18 yrs)	All-comers: HR allowed (19%; 8%; 30%)	Diarrhea 45%, neutropenia 30%; pneumonia 7.4%
**ALR** [45]**– NCT03863184**	Acalabrutinib	Rituximab	Lenalidomide	12 cycles → lenalidomide maintenance; stop with uMRD CR	24	64	19	2-yrs PFS 87.5%	2-yrs OS 95.7%	All-comers ( ≥ 18 yrs)	All-comers: HR allowed (23%; -; 64%)	Neutropenia: 38%; Rash: 42%; COVID-19 pneumonia: 25%
			**Risk-adapted strategy**									
**Study / NCT**	**BTKi**	**Anti-CD20**	**Third agent**	**Phase / design**	**n (pts)**	**Median age (yrs)**	**Follow-up (mo)**	**PFS**	**OS**	**Inclusion Criteria**	**Risk profile (TP53, blastoid/pleomorphic, Ki-67** ≥ **30%)**	**Most relevant Grade** ≥ **3 Toxicities (≥10%)**
**BOVEN** [46] **– NCT03824483**	Zanubrutinib	Obinutuzumab	Venetoclax	O × 38 cycles; Z + V ≥ 2 yrs	50	72	25	2-yrs PFS 86%	2-yrs OS 92%	Elderly (65 yrs of age or older)	High-risk with TP53 mutation	Neutropenia 10%; thrombocytopenia 8% and febrile neutropenia 8%
**Glove** [81] **– NCT03863184**	Glofitamab	Obinutuzumab	Lenalidomide	II, V from C1D1 plus O C1D15-16; Glofi step-up from C2D1 ×12 cycles; Len start C3-C12	25	65	13.1	6mo PFS 90%	6 mo OS 95%	All-comers ( ≥ 18 yrs)	High-risk (blastoid / TP53 / Ki-67 ≥ 50%/bulky disease/complex kayotype)	CRS 5%; neutropenia 23%
**WINDOW-3** [47] **– NCT03863184**	Acalabrutinib	Rituximab	Brexu-cel	II, AR × 9 cycles → brexu-cel ± A maintenance	20	61	13.1	1-yr PFS 94%	1-yr OS 100%	All-comers ( ≥ 18 yrs)	High-risk (blastoid / TP53 / Ki-67 ≥ 50%/bulky disease/complex kayotype)	CRS 15%; ICANS grade 3: 30% and grade 4: 15%

*A* Acalabrutinib, *ASCT* Autologous Stem Cell Transplantation, *BR* Bendamustine plus Rituximab, *BTKi* Bruton Tyrosine Kinase inhibitor, *C (e.g., C1D1)* Cycle (e.g., Cycle 1 Day 1), *CAR-T* Chimeric Antigen Receptor T-cell therapy, *CHOP* Cyclophosphamide, Doxorubicin, Vincristine, Prednisone, *CIT* Chemoimmunotherapy, *COVID-19* Coronavirus Disease 2019, *CR* Complete Response, *CRS* Cytokine Release Syndrome, *CT* Chemotherapy, *DHAP* Dexamethasone, High-dose Cytarabine, Cisplatin

*FFS* Failure-Free Survival, *GI* Gastrointestinal, *HR* High Risk, *I* Ibrutinib, *ICANS* Immune Effector Cell-Associated Neurotoxicity Syndrome, *IR* Ibrutinib plus Rituximab, *mo* Months, *MRD* Minimal Residual Disease, *mPFS* Median Progression-Free Survival

*NA* Not Available, *NCT* National Clinical Trial (ClinicalTrials.gov identifier number), *NR* Not Reached, *O* Obinutuzumab, *OR* Overall Response, *OS* Overall Survival, *PD* Progressive Disease, *PFS* Progression-Free Survival, *pts* Patients, *R* Rituximab, *R-CHOP* Rituximab plus CHOP, *R-DHAP* Rituximab plus DHAP, *uMRD* Undetectable Minimal Residual Disease, *V* Venetoclax,

*yrs* Years, *Z* Zanubrutinib.

## Relapsed/Refractory MCL

Ibrutinib has long been a major therapeutic option for R/R MCL patients [49–51]. However, the management of R/R MCL has changed as a result of its increasing use in frontline therapy and the advent of new drugs [38]. This evolution is particularly evident in the second-line setting, where an expanding range of therapeutic options is driving rapid changes in disease management (Fig. 1). As this evolution continues, differentiating between BTKi-naïve and BTKi-exposed patients will become even more crucial, as treatment history is likely to require distinct and more personalized therapeutic approaches in the R/R setting. The most relevant ongoing clinical trials and available treatment options, categorized by BTKi exposure, are summarized in (Tables 4–6).

**Table 4:** Selected clinical trials in R/R MCL.

				R/R MCL in BTKi-naïve patients								
Study / NCT	BTKi	Anti-CD20	Third Agent	Phase / Design	n	Median Age (yrs)	Follow-up (mo)	PFS (mo)	OS (mo)	Inclusion Criteria	Risk profile (TP53, blastoid, Ki-67)	Most relevant Grade ≥ 3 Toxicities (≥10%, per trial reports)
**SYMPATICO** [54] NCT03112174	Ibrutinib	Venetoclax	—	III, I ± V x 2 yrs → I until PD	267 (arm IV vs arm I- placebo: 134 vs 133)	71	51.2	mPFS, arm IV vs arm I-placebo: 31.9 vs 22.1 (p = 0.005)	mOS, arm IV vs arm I plus placebo: 44 vs 39 (p = 0.35)	All-comers (aged ≥18 yrs)	All-comers: HR allowed (arm IV vs arm I- placebo: 30%/28%; 13%/14%,-)	Neutropenia, thrombocytopenia, pneumonia, atrial fibrillation, hypertension, diarrhea (arm IV vs arm I- placebo: 31% vs 11%; 12% vs 11%; 5% vs 5%; 5% vs 9%)
					**R/R MCL in BTKi-exposed patients**							
**Study / NCT**	**BTKi**	**Anti-CD20**	**Third agent**	**Phase / design**	**n (pts)**	**Median age (yrs)**	**Follow-up (mo)**	**PFS (mo)**	**OS (mo)**	**Inclusion criteria**	**Risk profile (TP53, blastoid, Ki-67)**	**Most relevant Grade** ≥ **3 Toxicities (≥10%, per trial reports)**
**BRUIN** [57] NCT023740529	Pirtobrutinib	-	-	I/II, P until PD	152 (only cBTKi pre-treated pts)	70	17.5	mPFS 5.6	mOS 23.9	All-comers (aged ≥18 yrs)	All-comers: HR allowed – data not shown	Neutropenia 13%, infections 21%, atrial fibrillation, 3.6%
**ZUMA -2** [58] NCT02601313	Brexu-cel	-	-	II, LDP followed by brexu-cel	74 (apheresed) -68 (treated)	65	68	mPFS 25	mOS 47	All-comers (aged ≥18 yrs)	All-comers: HR allowed (17%, 31%, 82%); cBTKi-refractory 62%	Neutropenia 85%, anemia 50%, and thrombocytopenia 51%, infections in 32%. CRS 91% of pts (grade≥3, 15%), NEs in 63% (grade ≥3, 31%)
**TRANSCEND** [59] NCT02631044	Liso-cel	-	-	I/II, LDP followed by liso-cel	104 (apheresed)-83 (treated)	69	16	mPFS 15	mOS 18	All-comers (aged ≥18 yrs)	All-comers: HR allowed (23%, 31%, 75%); cBTKi-refractory 53%	Neutropenia 56%, anemia 37.5%, and thrombocytopenia 25%, infections in 15%, and prolonged cytopenia in 40%. CRS 61% of pts (grade 3/4, 1%; grade 5, 0), NEs in 31% (grade 3/4, 9%; grade 5, 0)

*BTKi* Bruton Tyrosine Kinase inhibitor, *cBTKi* Covalent Bruton Tyrosine Kinase inhibitor, *CRS* Cytokine Release Syndrome, *HR* High Risk, *I* Ibrutinib, *IV* Ibrutinib plus Venetoclax, *LDP* Lymphodepleting Chemotherapy, *MCL* Mantle Cell Lymphoma, *mo* Months, *mOS* Median Overall Survival, *mPFS* Median Progression-Free Survival, *NCT* National Clinical Trial identifier (ClinicalTrials.gov number), *NEs* Neurologic Events, *OS* Overall Survival, *PD* Progressive Disease, *PFS* Progression-Free Survival, *pts* Patients, *R/R* Relapsed/Refractory, *V* Venetoclax, *yrs* Years.

**Table 5:** Selected advanced/promising agents in clinical trials in MCL.

		Bispecific (anti-CD20xCD3) ± other agent										
Study / NCT	BTKi	Anti-CD20	Third Agent	Phase / Design	n (pts)	Median Age (yrs)	Follow-up (mo)	PFS (mo)	OS (mo)	Inclusion Criteria	Risk profile (TP53, blastoid/pleomorphic, Ki-67 ≥ 30%)	Most relevant Grade ≥ 3 Toxicities (≥10%)
**Glofitamab** [67] NCT03075696	— (BTKi-pretreated allowed)	Obinutuzumab (to mitigate CRS)	—	I/II, Glofi IV step-up: C1 (D8,D15) and from C2D1 ×12 cycles	60	72	19.6	mPFS: 16.7	mOS: 29.9	All-comers ( ≥ 18 yrs)	All-comers: HR allowed (8%, -, 46.7%)	neutropenia 23.3%, pneumonia 11.7%, anemia 11.7%, and CRS 11.7%
**Mosun-Pola** [71] NCT03671018	— (BTKi-pretreated allowed)	-	—	I, Mosun SC step-up: C1D1, C1D8, C1D15; then q3w from C2D1 for 17 cycles total. Pola: IV on C1–C6 D1.	20	71	7.2	-	-	All-comers ( ≥ 18 yrs)	All-comers; HR allowed (25%, 50%, 65%)	AEs (all grades): CRS 50%, ISR 50%, fatigue 45%, dyspnea 35%, paresthesia 30%, diarrhea 30%, myalgia 30%, IRR 25%, nausea 25%
				**ADC ± other agent**								
**Study / NCT**	**BTKi**	**Anti-CD20**	**Third Agent**	**Phase / Design**	**n (pts)**	**Median Age (yrs)**	**Follow-up (mo)**	**PFS (mo)**	**OS (mo)**	**Inclusion Criteria**	**Risk profile (TP53, blastoid/pleomorphic, Ki-67** ≥ **30%)**	**Most relevant Grade** ≥ **3 Toxicities (≥10%)**
**Lonca (LOTIS-2)** [72] NCT02669017	— (BTKi-pretreated allowed)	-	-	I/II, Loncastuximab q3w until PD	15	65	-	mPFS 4.8	mOS NR	R/R B-cell NHL; All-comers ( ≥ 18 yrs)	HR allowed; -	Neutropenia 39.7%, trombocitopenia 26.7%, liver toxicity 21.3%, anemia 15.3%
**Lonca + Ibrutinib (LOTIS-3)** [73] NCT03684694	Ibrutinib	-	-	I, Lonca q3w plus I up to 1 year	30	72 (DLBCL cohort median)	-	n.d. (ORR 62.2%, median DOR ~ 5.5 mo)	-	R/R B-cell NHL; All-comers ( ≥ 18 yrs)	HR allowed; -	Anemia 8.8%, thrombocytopenia/neutropenia ~5.9% each
**Lonca + Venetoclax** [74] NCT05053659	-	-	Venetoclax	I, Lonca q3w plus V x 6 cycles	13	66	-	-	-	R/R B-cell NHL; All-comers ( ≥ 18 yrs)	HR allowed; -	Grade ≥3 toxicity data not detailed (safety reported as manageable)
**Zilovertamab vedotin** [75] NCT03833180	-	-	-	I/II, ZV q3w until PD	15	68	-	-	-	R/R B-cell NHL; All-comers ( ≥ 18 yrs)	HR allowed; -	Grade ≥3 toxicity in neutrophils, hemoglobin, and platelets and as laboratory abnormalities; data not detailed (safety reported as manageable)
					**Biological combinations**							
**Study / NCT**	**BTKi**	**Anti-CD20**	**Third Agent**	**Phase / Design**	**n (pts)**	**Median Age (yrs)**	**Follow-up (mo)**	**PFS (mo)**	**OS (mo)**	**Inclusion Criteria**	**Risk profile (TP53, blastoid/pleomorphic, Ki-67** ≥ **30%)**	**Most relevant Grade** ≥ **3 Toxicities (≥10%)**
**AVO** [76] NCT04855695	Acalabrutinib	Obinutuzumab	Venetoclax	I/II, multi-cohort	26 (14 R/R, 12 TN)	68 (range 44-81)	14 (R/R) / 11 (TN)	71% (R/R) / 83% (TN) at 12 mo	90% (R/R) / 92% (TN) at 12 mo	R/R MCL after ≥1 anti-CD20 therapy (cohort A); TN, treatment-naïve, TI or TP53-aberrant (cohort B)	All-comers: HR allowed (TP53-aberrant 43% (R/R), 83% (TN); Blastoid 21% (R/R), 8% (TN); Ki-67 ≥ 30% 58% (TN))	Neutropenia 20%, Thrombocytopenia 12%, Anemia 8%, Infections 16%
**VIPOR** [76] NCT03223610	Ibrutinib	Obinutuzumab	Venetoclax, Prednisone, Lenalidomide	Phase 1b → Phase 2, fixed-duration 6 cycles (q21d), no maintenance	36 (16 R/R, 20 TN)	67	24.1	24mo-PFS 95%	24mo-OS 100%	R/R or treatment-naïve MCL, age >18, adequate organ function; prior therapy allowed (excluding prior VEN or lenalidomide), prior BTKi allowed in some R/R	All-comers: HR allowed (TP53 mutation/deletion 32%; TP53 overexpression >50% 19%; blastoid morphology 25%; Ki-67 > 30% 37%)	thrombocytopenia 14%, neutropenia 13%, anemia 10% (per cycles) — no febrile neutropenia; non-hematologic: hypokalemia (any grade 89%), diarrhea 67%, rash 58%; G3 non-heme AEs ≥10%: hypokalemia 25%, rash 11%; no TLS, no treatment-related mortality
						**Small molecules**						
**Study / NCT**	**BTKi**	**Anti-CD20**	**Third Agent**	**Phase / Design**	**n (pts)**	**Median Age (yrs)**	**Follow-up (mo)**	**PFS (mo)**	**OS (mo)**	**Inclusion Criteria**	**Risk profile (TP53, blastoid/pleomorphic, Ki-67** ≥ **30%)**	**Most relevant Grade** ≥ **3 Toxicities (≥10%)**
**Sonrotoclax plus Zanubrutinib** [78] NCT04277637	Zanubrutinib	-	Sonrotoclax	I, multi-cohort	35	68	16.4	24 mo-DOR rate 84%	-	R/R MCL after ≥1 anti-CD20 therapy.	All-comers: HR allowed	Neutropenia 20%, Thrombocytopenia 12%, Pneumonia 12%

*ADC* Antibody–Drug Conjugate, *AEs* Adverse Events, *AVO* Acalabrutinib, Venetoclax, Obinutuzumab combination, *BTKi* Bruton Tyrosine Kinase inhibitor, *C (e.g., C1D1)* Cycle (e.g., Cycle 1 Day 1), *CRS* Cytokine Release Syndrome, *DLBCL* Diffuse Large B-Cell Lymphoma, *DOR* Duration of Response, *Glofi* Glofitamab, *G3* Grade 3, *HR* High Risk, *IRR* Infusion-Related Reaction, *ISR* Injection-Site Reaction, *IV* Intravenous, *LOTIS* Loncastuximab Tesirine clinical trial program, *MCL* Mantle Cell Lymphoma, *mo* Months, *Mosun* Mosunetuzumab, *Mosun-Pola* Mosunetuzumab plus Polatuzumab vedotin, *mOS* Median Overall Survival, *mPFS* Median Progression-Free Survival, *NCT* National Clinical Trial identifier (ClinicalTrials.gov number), *NHL* Non-Hodgkin Lymphoma, *NR* Not Reached, *n.d.* Not Determined, *ORR* Overall Response Rate, *OS* Overall Survival, *PD* Progressive Disease, *PFS* Progression-Free Survival, *Pola* Polatuzumab vedotin, *pts* Patients, *q3w* Every 3 weeks, *q21d* Every 21 days, *R/R* Relapsed/Refractory, *SC* Subcutaneous, *TI* Treatment-Intolerant, *TLS* Tumor Lysis Syndrome, *TN* Treatment-Naïve *VEN / V* Venetoclax, *VIPOR* Venetoclax, Ibrutinib, Prednisone, Obinutuzumab, Lenalidomide regimen, *ZV* Zilovertamab vedotin *yrs* Years.

**Table 6:** Selected ongoing phase II-III trials in MCL.

Untreated MCL – BTKi-naïve patients					
BTKi-based combinations					
*Including anti-CD20 monoclonal antibody +/- CIT*					
NCT / EU-CT	Phase	n	Regimen	Key inclusion criteria	High-risk allowed (TP53 / blastoid / Ki-67 ≥ 30%)
NCT05976763	III	421	Zanubrutinib + Rituximab	Older patients; all-comers	Not specified
NCT05004064	II	48	Acalabrutinib + Rituximab	All-comers	Not specified
NCT04566887	II	105	Acalabrutinib + R-CHOP	Fit, chemo-eligible	Not specified
NCT04802590	II	194	Ibrutinib + anti-CD20 + Venetoclax	All-comers	Not specified
NCT06263491	II	50	Pirtobrutinib + Rituximab	Low–intermediate MIPI only	No (high-risk excluded)
NCT06572618	II	28	Nemtabrutinib + Rituximab	All-comers	Not specified
NCT04736914	II	47	R-CHOP/R-DHAOx + Zanubrutinib → ASCT → Zanubrutinib maint.	Young/fit, transplant-eligible	Not specified
NCT06522386	II	40	Pirtobrutinib + Rituximab + Venetoclax	All-comers	Not specified
NCT06846489	II	50	Acalabrutinib + Rituximab	All-comers	Not specified
NCT04883437	II	49	Acalabrutinib + Obinutuzumab	All-comers	Not specified
NCT04855695	II	50	Acalabrutinib + Venetoclax + Obinutuzumab	All-comers	Yes (TP53-aberrant allowed)
EU-CT 2022-501808-96-00	II	150	Venetoclax + Ibrutinib + Rituximab vs BR + Ibrutinib + Rituximab	All-comers	Not specified
*Including anti-CD20 bispecific antibodies*					
NCT06558604 (GLOASIS)	II	100	Glofitamab + Venetoclax ± Zanubrutinib	All-comers	Not specified
NCT06357676	II	27	Glofitamab + Ibrutinib + Obinutuzumab	All-comers	Not specified
*Including small molecules*					
NCT03523975	II	28	Venetoclax + Lenalidomide + Rituximab	All-comers	Not specified
*Including small molecules or CAR-T cells*					
NCT06482684	II	150	Brexu-cel after short induction (R + Ibrutinib)	Fit, cellular-therapy eligible	Yes
**Relapsed / Refractory (R/R) MCL**					
**BTKi-based combinations**					
**NCT**	**Phase**	**n**	**Regimen**	**Key inclusion criteria**	**High-risk allowed**
NCT06742996	III	300	Sonrotoclax + Zanubrutinib vs placebo	R/R, BTKi-naïve	Not specified
NCT05529069	II	30	Venetoclax + Pirtobrutinib	Prior BTKi	Not specified
NCT04855695	II	50	Acalabrutinib + Venetoclax + Obinutuzumab	Frontline & R/R	Yes
*Including anti-CD20 bispecific antibodies*					
NCT06084936	III	182	Glofitamab vs investigator’s choice	Prior BTKi	Yes
NCT06252675	II	30	Glofitamab + Pirtobrutinib	Prior BTKi	Yes
NCT06054776	II	40	Acalabrutinib + Obinutuzumab + Glofitamab	Prior BTKi	Yes
NCT06558604	II	100	Glofitamab + Venetoclax ± Zanubrutinib	Prior BTKi	Yes
*Including CAR-T cells*					
NCT04484012	II	30	CD19 CAR-T + Acalabrutinib	Prior BTKi	Yes
NCT06553872	II	60	Pirtobrutinib + Brexucabtagene autoleucel	Prior BTKi	Yes
**ADCs based combinations**					
NCT05249959	II	49	Loncastuximab + R-BAC	Prior BTKi	Yes
NCT05868395	II	40	Polatuzumab + BR	Prior BTKi	Yes

*ADC* antibody–drug conjugate, *ASCT* autologous stem cell transplantation, *BR* bendamustine plus rituximab, *BTKi* Bruton Tyrosine Kinase inhibitor, *CAR-T* chimeric antigen receptor T cells, *CR* complete response, *CRS* cytokine release syndrome, *DOR* duration of response, *EU-CT nr* European Clinical Trial number, *HR* high-risk, *IV* intravenous, *MCL* mantle cell lymphoma, *MIPI* Mantle Cell Lymphoma International Prognostic Index, *n.d.* not determined, *NCT nr* National Clinical Trial number *NR* not reached, *ORR* overall response rate, *OS* overall survival, *PFS* progression-free survival, *Pts* patients, *q3w* every 3 weeks, *R-CHOP* rituximab, cyclophosphamide, doxorubicin, vincristine, and prednisone, *R-DHAOx* rituximab, dexamethasone, high-dose cytarabine, and oxaliplatin, *R/R* relapsed/refractory, *R-BAC* rituximab + bendamustine + cytarabine, *SC* subcutaneous, *TN* treatment-naïve, *TP53* tumor protein p53.

### R/R MCL in BTKi-naïve patients

As BTK inhibitors and targeted regimens become increasingly incorporated into frontline therapy, the population of patients who are truly BTKi-naïve in relapse is expected to shrink.

Despite the substantial efficacy observed with BTKi monotherapy [52], strategies to further deepen and prolong responses are actively being explored. In this context, ibrutinib plus venetoclax (I + V) may find a role primarily as a re-treatment strategy for patients who achieve a long remission after initial therapy [53]. Recent clinical findings indicate that this combination may enhance therapeutic responses and optimize disease management relative to BTKi monotherapy, while preserving an acceptable safety profile [54]. However, its optimal positioning remains unclear, particularly as third-generation BTK inhibitors and other targeted combinations begin to shift the first-line paradigm.

### R/R MCL in BTKi-exposed patients

Patients with R/R MCL who have been exposed to BTKi represent a challenging population, as resistance or intolerance can severely constrain subsequent treatment options [55, 56]. For such patients, the development of novel strategies, including the use of non-covalent BTKi [57] or cellular therapies [58, 59] constitute a critical priority. An additional emerging population includes patients who relapse following a fixed-duration BTKi therapy after a period off treatment. Currently no clear indications exist for this group; however, rechallenge with BTKi alone or in combination may be a reasonable option in cases of late relapses [38].

For patients who relapse after completing a defined course of BTKi therapy, as well as for those who experience disease progression while receiving continuous BTKi treatment, choosing the most suitable therapeutic approach requires careful, individualized evaluation. In the past, options, such as immunochemotherapy combinations [60] and agents like venetoclax [61] have been used, but their efficacy has generally been modest, and their role in current practice remains quite restricted.

More recently, non-covalent BTKi, such as pirtobrutinib [57] and cellular therapies like chimeric antigen receptor (CAR) T-cells [58, 59] have shown activity after BTKi failure, offering new perspectives for R/R MCL patients. By contrast, allogeneic stem cell transplantation (allo-SCT) now plays a more limited role due to its associated toxicity and the availability of more effective and targeted strategies [62].

#### Conventional approaches – Chemoimmunotherapy

The role of conventional CIT in BTKi-R/R MCL is increasingly limited [55, 56]. Overall, its efficacy is modest, and its use is likely to be confined to bridging patients to cellular therapies or to those few who cannot access targeted agents.

Rituximab-bendamustine-cytarabine (R-BAC) is an exception, showing meaningful activity even in patients previously treated with BTK inhibitors [60]. However, while initial responses can be high, durability is often limited [60], highlighting the need for effective consolidation strategies. Ongoing studies, such as the FIL-COLUMN trial (EudraCT number: 2021-000715-23), aim to address this gap (see the Future perspective-immunoconjugates section below for more details).

In clinical practice, CIT will likely remain a niche option in R/R MCL. The broader trend favors targeted therapies and immunoconjugates, reflecting the shift toward more effective, less toxic approaches that can provide durable disease control while minimizing treatment-related morbidity.

#### Noncovalent BTKi – Pirtobrutinib

Pirtobrutinib (LOXO-305) is a next-generation, non-covalent BTK inhibitor designed for patients with relapsed or refractory MCL who have developed resistance or intolerance to first- and second-generation covalent BTK inhibitors. By reversibly binding BTK, pirtobrutinib can overcome resistance mutations that limit the efficacy of earlier agents, offering a unique mechanism of action [57].

Clinical experience from the BRUIN trial indicates that pirtobrutinib can induce meaningful responses with a favorable safety profile, including minimal cardiovascular or bleeding toxicity [57, 63]. Common side effects, such as fatigue, diarrhea, and mild gastrointestinal, or musculoskeletal symptoms, are generally manageable [57, 63], making it suitable for older patients or those with comorbidities.

In practice, pirtobrutinib may although have a role as a bridge to CAR-T therapy or as an option for patients who cannot access cellular therapies. It also provides a salvage pathway for patients who fail covalent BTK inhibitors. However, its use is not straightforward: outcomes in high-risk patients remain modest, and its optimal positioning in the evolving treatment landscape, where newer therapies are increasingly available, remains uncertain.

#### Anti-CD19 CAR T-cell therapy

Autologous anti-CD19 CAR T-cell therapies have dramatically reshaped the treatment landscape for patients with R/R MCL, offering a level of efficacy that was previously unattainable with conventional therapies (Fig. 1) [58, 59]. Brexucabtagene autoleucel (brexu-cel, KTE-X19) became the first FDA-approved CAR-T product for MCL in 2020, following the pivotal ZUMA-2 trial [58]. This study demonstrated high early response rates, with an ORR of 91% and a complete response rate of 68% among treated patients. Importantly, these impressive responses were observed across multiple clinical subgroups, including older patients and those with high-risk disease features, underscoring the broad applicability of this approach [58]. Subsequent real-world studies have largely confirmed the efficacy and safety outcomes seen in the clinical trial setting [64–66], reinforcing the notion that CAR-T therapy can induce rapid and meaningful remissions in a population historically characterized by limited treatment options and poor prognosis.

Despite these encouraging early results, the durability of remissions remains a central challenge. Certain high-risk features, such as *TP53* mutations, elevated Ki-67 proliferation indices, and prior exposure to bendamustine, are associated with shorter remission durations and a higher likelihood of relapse [65]. While CAR-T therapies provide profound early disease control, long-term follow-up data indicate that relapses can still occur, highlighting the need for careful patient monitoring and potential consolidation or maintenance strategies to prolong remission. The safety profile, although generally manageable [64–66], requires significant attention. Cytokine release syndrome (CRS) and neurotoxicity remain the most clinically significant adverse events. Most CRS events are mild to moderate and can be effectively managed with supportive care, including the IL-6 receptor antagonist tocilizumab, but severe cases may necessitate intensive care and careful monitoring [65]. In addition, infections remain a notable concern due to lymphodepleting conditioning and therapy-related immunosuppression, requiring vigilance and appropriate prophylaxis.

Lisocabtagene maraleucel (liso-cel), approved in 2021, has offered a complementary CAR-T option, demonstrated similarly high early response rates (ORR 83%, CR 72%) while exhibited generally lower rates of severe CRS and neurotoxicity [59]. These favorable safety outcomes are likely related to its defined T-cell composition and streamlined manufacturing process. Preliminary data suggest that liso-cel may achieve comparable efficacy to brexu-cel with improved tolerability, making it an attractive option for patients at higher risk of treatment-related toxicity. However, as with brexu-cel, longer follow-up and real-world evidence are required to fully assess durability, late adverse effects, and optimal patient selection.

In clinical practice, CAR-T therapies have emerged as a powerful and transformative option for patients with aggressive or multiply relapsed MCL [28]. The integration of CAR-T into routine clinical practice necessitates careful patient selection, logistical planning, and multidisciplinary coordination. Overall, while CAR-T represents a major advancement in the care of R/R MCL, ongoing research and longer-term follow-up will be essential to define its full potential, optimize outcomes, and determine how best to combine or sequence these therapies with other novel targeted strategies.

#### Allogenic stem cell transplantation

Allogenic stem cell transplantation (Allo-SCT) remains a potentially curative option for selected MCL patients, particularly those with high-risk features or early relapses after initial therapy [38]. This approach is especially relevant for individuals who experience disease recurrence following CAR T-cell therapy failure. However, with the increasing availability of novel agents, the role of allo-SCT has become more restricted and is now generally reserved for patients with multiple relapses or refractory disease who have exhausted alternative therapeutic options [62].

### Future perspectives in R/R MCL – novel agents under investigation

#### Bispecific antibodies

Bispecific antibodies (bsAbs) represent a rapidly evolving therapeutic class in R/R MCL (Fig. 1) [67]. By simultaneously engaging CD3-positive T cells and CD20-expressing malignant B cells, bsAbs offer an “off-the-shelf” immunotherapeutic approach that circumvents the logistical constraints of CAR T-cell therapy. This characteristic makes them particularly attractive for older or comorbid patients who may not be candidates for cellular therapy, a population that continues to expand as frontline and second-line treatment strategies evolve.

Despite their strong biological rationale, the clinical role of bsAbs in MCL remains incompletely defined. Current evidence is largely derived from early-phase trials [67], and their integration within existing treatment algorithms, dominated by BTK inhibitors and CAR-T, requires further clarification. However, bsAbs are emerging as a promising option in two clinically relevant settings: patients relapsing after CAR T-cell therapy, where therapeutic options are limited and outcomes remain poor; and patients progressing after covalent BTK inhibition who are ineligible for CAR T cells, either due to age, failure, or medical comorbidities.

Among the agents under investigation, glofitamab is the most advanced. Early studies have shown meaningful activity in heavily pretreated MCL, including BTKi- and CAR-T–exposed disease, with rapid and sometimes deep responses [67]. The safety profile appears manageable, with cytokine release syndrome being the most frequent adverse event but typically low grade and responsive to standard management. Neurotoxicity is less common than in CAR-T recipients, although infection risk, particularly early during treatment, remains a concern [67]. The ongoing phase III GLOBRYTE trial will be pivotal in defining the therapeutic positioning of glofitamab relative to standard options [68].

Other CD20×CD3 bsAb, such as epcoritamab [69], odronextamab [70], and emerging combination strategies (e.g. mosunetuzumab plus polatuzumab [71]) are also showing early signs of activity in R/R MCL, though their development is less advanced. Whether these agents will offer distinct advantages over glofitamab or find a niche in specific biological subgroups or post–cellular therapy relapse remains to be determined.

Overall, while bsAbs are poised to become an important addition to the therapeutic armamentarium for R/R MCL, their optimal positioning remains unsettled. They are particularly promising for patients who fail CAR-T or cannot access or tolerate cellular therapy after BTKi failure, but long-term data and randomized studies are still needed. As the treatment landscape continues to shift with next-generation BTK inhibitors, CAR-T refinements, and evolving frontline strategies, the precise role of bsAbs will likely depend on future evidence regarding durability, sequencing, and patient selection.

#### Immunoconjugates

Antibody–drug conjugates (ADCs) represent another targeted strategy under investigation for R/R MCL (Fig. 1). By coupling a monoclonal antibody with a potent cytotoxic payload, ADCs aim to deliver chemotherapy selectively to malignant B cells while limiting systemic exposure [72]. Although biologically compelling, their clinical development in MCL is less advanced than for bispecific antibodies, and emerging data suggests that their ultimate therapeutic role may be more restricted, largely serving highly pretreated patients who are not candidates for BTK inhibitors, CAR T-cell therapy, or bispecific antibodies.

Loncastuximab tesirine (Lonca), a CD19-directed ADC, has provided the most substantive early signals. Small MCL subsets within broader studies, such as LOTIS-2, have shown that Lonca can induce meaningful responses, including in patients previously treated with BTK inhibitors [72]. Toxicities, dominated by cytopenias, liver enzyme elevations, and edema, are generally manageable but require careful monitoring and dose adjustments [72]. While these findings suggest on-target activity, durability of responses remains uncertain, and its use is likely to remain constrained to later lines of therapy.

Combination approaches may enhance the utility of Lonca. Preclinical and early studies suggest synergy with BTK inhibitors [73], conventional CIT, and BCL-2 inhibition [74], offering potential avenues to improve depth and duration of response. The FIL_COLUMN trial (EudraCT number 2021‑000715‑23) is currently exploring Lonca as a consolidation strategy after short-term R-BAC in BTKi-pretreated MCL, a setting where conventional options are limited and often poorly tolerated.

Another emerging agent, zilovertamab vedotin, targets ROR1, a tumor-associated antigen with minimal expression in normal adult tissues. Early-phase studies have reported encouraging activity in heavily pretreated B-cell malignancies, including MCL after BTKi failure, with an acceptable toxicity profile [75]. However, these data remain preliminary, and its precise role, whether as monotherapy or in combination, will depend on ongoing trial results.

Taken together, ADCs provide an additional targeted modality for R/R MCL, but current evidence suggests their future positioning may be more niche than that of bispecific antibodies or novel BTK inhibitors. They are most likely to benefit patients who have exhausted BTK inhibition, are not candidates for CAR-T therapy, and may not tolerate or access T-cell–redirecting therapies. As clinical experience expands, ADCs may find a defined place within tailored therapeutic sequences, but their overall contribution is expected to be more complementary than transformative in the evolving MCL landscape.

#### Biological combinations

Chemotherapy-free regimens are an increasingly explored option in R/R MCL, aiming to improve outcomes while reducing treatment-related toxicity. BTK-based combinations, such as the AVO regimen (acalabrutinib, venetoclax, obinutuzumab), have emerged as particularly promising. In this investigator-initiated, multicenter, multi-cohort phase I/II trial, AVO demonstrated high rates of complete responses and MRD negativity in both R/R and a small frontline cohort, including patients with *TP53*-aberrant disease [75]. While these early results highlight the biological potency of multi-agent BTK strategies, follow-up remains short, and the durability of responses, long-term safety, and optimal positioning of AVO in the frontline or relapsed MCL landscape are still to be defined.

The ViPOR combination—venetoclax, ibrutinib, prednisone, obinutuzumab, and lenalidomide—has generated considerable interest. In a small, single-center phase Ib/II trial, ViPOR delivered impressive early activity, with high rates of undetectable MRD and complete responses after six fixed-duration cycles [76, 77]. These results highlight the biological potency of this multi-agent target strategy. However, evidence remains extremely limited, and the durability of responses, real-world tolerability, and applicability across broader patient populations are still unclear.

#### Small molecules

Among emerging small-molecule agents, sonrotoclax, a next-generation oral BCL2 inhibitor, has gained attention for R/R MCL. Early clinical data suggest meaningful activity with a favorable pharmacologic profile, leading to FDA Fast Track designation [78]. A pivotal phase III study comparing sonrotoclax plus zanubrutinib versus zanubrutinib alone in previously treated BTKi-naïve or BTKi-intolerant patients is underway; until these results become available, the true added value of sonrotoclax remains uncertain.

Beyond BCL2 inhibition, several novel agents aim to improve upon existing BTKi therapy by achieving deeper and more sustained target suppression. BTK degraders, such as BGB-16673, represent one of the most innovative approaches, capable of eliminating BTK rather than merely inhibiting it. Early phase I indicate activity even in patients harboring resistance mutations to both covalent and non-covalent BTKi [79], but clinical experience remains very limited, and the long-term safety profile is largely unknown.

Golcadomide, an oral cereblon E3 ligase modulator, is another small molecule under exploration, currently evaluated in combination with R-CHOP in aggressive B-cell lymphomas. While preliminary signals suggest anti-tumor activity with manageable toxicity, its specific utility in MCL is still speculative [80].

Overall, these novel small molecules expand the pipeline of targeted strategies for R/R MCL, but their role is far from defined. Compared with bispecific antibodies or CAR-T therapies, which are further ahead in development, most of these agents will likely occupy niche positions until robust data from larger, multicenter trials clarify their real clinical impact.

## Conclusion

**Fig. 2:**
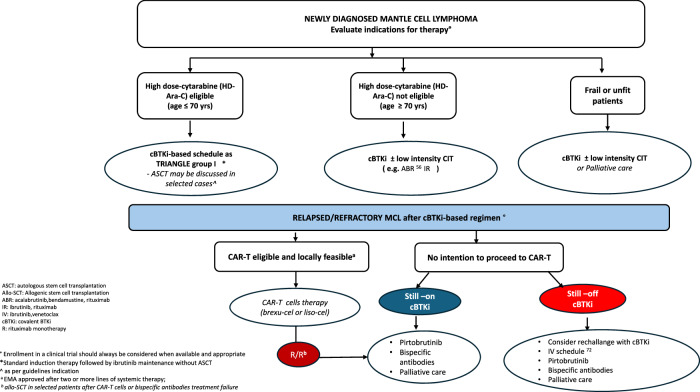
Schematic representation of the new algorithmic enhancements applied to the MCL process. ASCT autologous stem cell transplantation, Allo-SCT: Allogenic stem cell transplantation, ABR: acalabrutinib bendamustine rituximab, IR ibrutinib rituximab, IV: ibrutinib venetoclax, cBTKi covalent BTKi, R rituximab monotherapy, ° Enrollment in a clinical trial should always be considered when available and appropriate, *Standard induction therapy followed by ibrutinib maintenance without ASCT, ^ as per guidelines indication, anot formally EMA approved, bEMA approved after two or more lines of systemic therapy.

The therapeutic management of MCL is undergoing a rapid transformation, leading to substantial improvements in patient outcomes. Central to this evolution is the growing emphasis on biologically driven therapies, including BTKi and cellular- or immune-based therapies, which aim to deliver durable disease control, reduce treatment-related toxicities, and redefine therapeutic strategies (Fig. 2).

A critical trajectory in this shifting paradigm is the development of rational combination regimens specifically designed to overcome resistance, enhance response rates, and achieve long-term remission, even among high-risk or heavily pretreated patients. In parallel, the broader adoption of genomic profiling and molecularly targeted therapeutics is expected to further refine treatment algorithms, paving the way for increasingly personalized clinical decision-making.

Equally important will be the role of well-designed real-world studies to confirm the benefits observed in clinical trials, assess the generalizability of novel regimen, and capture both clinician and patient perspectives. In this context, systematic evaluation of QoL outcomes should be prioritized to ensure that therapeutic advances translate into meaningful patient benefits.

Ultimately, the full potential of emerging MCL treatments will depend on timely and equitable access to these innovations worldwide. Overcoming geographic and economic barriers to care is essential to ensure that the remarkable progress achieved in recent years translates into improved outcomes for all patients with MCL.
